# Interaction of Human Tumor Viruses with Host Cell Surface Receptors and Cell Entry

**DOI:** 10.3390/v7052592

**Published:** 2015-05-22

**Authors:** Georgia Schäfer, Melissa J. Blumenthal, Arieh A. Katz

**Affiliations:** 1 Institute of Infectious Disease and Molecular Medicine, Faculty of Health Sciences, University of Cape Town, Observatory 7925, South Africa; E-Mails: BLMMEL001@myuct.ac.za (M.J.B.); arieh.katz@uct.ac.za (A.A.K.); 2 Division of Medical Biochemistry, Faculty of Health Sciences, University of Cape Town, Observatory 7925, South Africa; 3 MRC/UCT Receptor Biology Research Unit, Faculty of Health Sciences, University of Cape Town, Observatory 7925, South Africa

**Keywords:** oncogenic viruses, host cell receptors, viral attachment, entry mechanisms

## Abstract

Currently, seven viruses, namely Epstein-Barr virus (EBV), Kaposi’s sarcoma-associated herpes virus (KSHV), high-risk human papillomaviruses (HPVs), Merkel cell polyomavirus (MCPyV), hepatitis B virus (HBV), hepatitis C virus (HCV) and human T cell lymphotropic virus type 1 (HTLV-1), have been described to be consistently associated with different types of human cancer. These oncogenic viruses belong to distinct viral families, display diverse cell tropism and cause different malignancies. A key to their pathogenicity is attachment to the host cell and entry in order to replicate and complete their life cycle. Interaction with the host cell during viral entry is characterized by a sequence of events, involving viral envelope and/or capsid molecules as well as cellular entry factors that are critical in target cell recognition, thereby determining cell tropism. Most oncogenic viruses initially attach to cell surface heparan sulfate proteoglycans, followed by conformational change and transfer of the viral particle to secondary high-affinity cell- and virus-specific receptors. This review summarizes the current knowledge of the host cell surface factors and molecular mechanisms underlying oncogenic virus binding and uptake by their cognate host cell(s) with the aim to provide a concise overview of potential target molecules for prevention and/or treatment of oncogenic virus infection.

## 1. Introduction

Cell entry is a fundamental process of the infectivity of any virus, and cell surface receptors are the critical molecules in target cell recognition determining cell tropism and species specificity [1,2]. To date, there are seven known human oncogenic viruses, namely Epstein-Barr virus (EBV), Kaposi’s sarcoma-associated herpes virus (KSHV), high-risk human papillomaviruses (HPVs), Merkel cell polyomavirus (MCPyV), hepatitis B virus (HBV), hepatitis C virus (HCV) and human T cell lymphotropic virus type 1 (HTLV-1). These viruses belong to distinct viral families, display different modes of entry and host cell tropism and lead to distinct types of cancer. They all infect their specific target cells through a two-step process: the first step involves attachment to the cell surface followed by the second step of specific receptor-mediated uptake. With the exception of EBV [3] which uses the cellular receptor CD21 (or CR2) for attachment [4], all oncogenic viruses initially attach to their target cells via ubiquitously expressed cell membrane heparan sulfate proteoglycans (HSPG), also termed the “universal receptor”, which contain charged carbohydrate moieties that can interact with several viral surface glycoproteins or protein ligands expressed in the envelope and/or the capsid, respectively. This leads to accumulation of the viral particle at the cell surface and is generally followed by conformational changes ultimately facilitating viral uptake by secondary cell type- and/or virus-specific receptors. Some oncogenic viruses display simple binding and uptake involving only a small number of participating viral and/or host surface molecules, while others involve multiple viral proteins engaging with several specific receptors, co-receptors and co-factors that may vary according to cell type, implicating rather complex, highly regulated and concerted interactions [1,2].

This review summarizes the virus and host cell surface molecular interactions essential for productive viral infection and initiation of the viral life cycle of the individual oncogenic viruses that culminate in the tumorigenic process. While human immunodeficiency virus (HIV) is not considered a tumor virus *per se*, immunodeficiency-related malignancies play an important role in the total number of oncogenic virus-associated cancer types and will be mentioned where appropriate. 

## 2. Epstein-Barr Virus

Epstein-Barr virus (EBV) belongs to the large and diverse family of *Herpesviridae* (subfamily Gammaherpesvirinae). As the first virus that has been identified as an etiologic agent for human cancer, EBV is now known to be causally associated with several B cell malignancies including Burkitt’s lymphoma, Hodgkin’s lymphoma, immunosuppression-related lymphoma, T and NK cell lymphomas as well as malignancies involving epithelial cells of the upper digestive tract, particularly nasopharyngeal and stomach carcinomas [5]. Found ubiquitously in approximately 95% of the adult population worldwide, most individuals acquire EBV infections during early childhood when EBV establishes a latent infection that persists generally asymptomatically throughout life. However, under certain circumstances when host-virus balance is not achieved such as due to immunosuppression as a result of HIV infection or in response to unrelated infections, EBV can cause malignant diseases [6]. As a predominantly orally transmitted virus, EBV has (unlike other members of the herpesvirus family) a rather narrow spectrum of potential target cells and primarily infects naïve tonsillar B cells [7] and (more rarely) oral epithelial cells [8]. 

Like all members of the herpesvirus family, the comparatively large EBV double-stranded linear DNA genome is packed inside a capsid which is surrounded by a tegument. This is further enclosed by a lipid envelope consisting of several conserved, as well as EBV-unique, glycoproteins. These glycoproteins play important roles during initial attachment and subsequent viral entry through interaction with specific host cell surface receptors mediating macropinocytosis [9] and lipid raft-dependent endocytosis [9,10]. It has long been known that the initial phase of EBV tethering to the host cell surface of B cells and epithelial cells occurs via the viral envelope glycoprotein gp350 (or its alternative isoform gp220) which interacts with the cellular receptor CD21 (or CR2) [4]. A recent report suggested also the involvement of CD35 as an alternative EBV attachment receptor in certain CD21-negative cells [11]. Unique among herpesviruses, gp350/220 dominates the outer viral membrane and is one of the most abundant EBV glycoproteins [12]. Although the absence of gp350/220 reduces EBV entry into epithelial and B cells, it is not absolutely required for infection [13]. In addition, the EBV transmembrane envelope glycoprotein BMRF2 has been shown to interact with β1 and α5 integrins on oral epithelial cells but not on B cells [14]. 

Initial tethering of EBV to either the B cell or the epithelial cell membrane eventually triggers fusion with the EBV envelope which is considered the second phase of the infection process. This requires three conserved viral glycoproteins comprising the core fusion machinery, namely the gH-gL heterodimer and gB, the latter being crucial to the fusion process as it can insert into target membranes and refold through large conformational changes to bring viral and host membranes into close proximity, resulting in the formation of a fusion pore [15]. However, activation of this core fusion machinery differs significantly for each cell type [15]. While infection of B cells occurs mainly via endocytosis followed by fusion of the virus envelope with the endocytic vesicle membrane, epithelial cells are generally entered through direct fusion with the host cell plasma membrane at the cell surface [15]. In B lymphocytes, it was found that EBV uses the host cell surface human leukocyte antigen class II (HLA class II) through binding to the viral glycoprotein gp42 which associates non-covalently with the complex of the core fusion machinery gH-gL and gB. This interaction eventually triggers fusion of the virus with the endosomal membrane, allowing entry of the tegumented capsid into the cytoplasm [16]. While the interaction between gp42, gH-gL and gB is required for EBV entry into B cells, infection of epithelial cells requires only gH-gL and gB [17]. In fact, gp42 is not only dispensable but was even found to be inhibitory for epithelial cell infection [17]. EBV fusion with the plasma membrane and entry into epithelial cells has recently been shown to involve neuropilin 1 (NRP1) which directly interacts with gB [9], as well as binding of gH-gL to αVβ6 and αVβ8 integrins [18] via recruitment and activation of receptor tyrosine kinases and signaling via the epidermal growth factor receptor (EGFR)/Akt and EGFR/extracellular-signal-regulated kinases (ERK) pathways [9]. The presence or absence of gp42 in the EBV envelope renders the virus mutually exclusive for B cell and epithelial cell entry, respectively, therefore pointing towards the role of gp42 in determining (and redirecting) EBV’s cell tropism [17]. 

## 3. Kaposi’s Sarcoma-Associated Herpes Virus

Kaposi’s sarcoma-associated herpes virus (KSHV) or human herpesvirus-8 (HHV-8) is a gamma-2 or rhadinovirus subfamily herpesvirus (*Herpesviridae* family) that causes two types of malignancies: the vascular tumor known as Kaposi’s Sarcoma (KS), including classical KS, African-endemic KS, iatrogenic KS and epidemic or AIDS-associated KS, as well as B cell malignancies including multicentric Castleman disease and primary effusion lymphoma [19,20,21]. With the onset of the AIDS epidemic, immunodeficiency-related neoplasias have been steadily on the rise, and KS is now considered the most common AIDS-related malignancy worldwide [22,23,24]. Unlike EBV, KSHV is not a ubiquitous virus but is most prevalent in African populations [25]. 

KSHV DNA sequences can be detected in various body fluids such as blood, plasma, semen, and saliva. However, transmission of the infection seems to mainly occur through contaminated saliva [26,27,28,29,30,31]. KSHV infects a variety of target cells, such as activated B cells, endothelial cells, monocytes (including macrophages and dendritic cells), fibroblasts and epithelial cells [32].

As a member of the herpesvirus family, KSHV structure shows close similarities to EBV as described above, with the KSHV double-stranded linear DNA genome packed inside a capsid which is surrounded by a tegument and further enclosed by a lipid envelope, the latter consisting of five conserved glycoproteins, namely gB, gH, gL, gM and gN, as well as several KSHV-unique lytic cycle associated glycoproteins such as ORF4 and gpK8.1A [32,33]. Although KSHV displays broad cell tropism with the available host cell surface molecules determining the particular cell-type specific mode of entry via engagement with multiple KSHV glycoproteins [34], generally, glycoproteins gB and gpK8.1A function as key molecules in the initial attachment of the virus to the cell surface [35], while the non-covalently linked gH-gL complex is indispensable for subsequent KSHV entry [36]. 

In the initial phase of the infectious cell entry, KSHV attaches to host cell surface HSPGs mostly via gB and gpK8.1A [35,37], possibly followed by conformational changes in viral glycoproteins allowing access to specific entry receptors which function in the second phase of the infection [38]. According to cell tropism and entry pathway, these entry receptors vary and/or are utilized by KSHV in different combinations. Several studies on human fibroblasts and human endothelial cells demonstrated the necessity of viral gB engagement with the integrins α3β1, αVβ3, αVβ5 for cell entry [39,40], and recently also the interaction of gB with integrin α9β1 has been discussed [41]. Multiple integrin receptors can interact with the glutamate/cystine exchange transporter xCT, which has been identified as a fusion-entry receptor mediating KSHV-induced endocytic events [39,42]. However, it is yet unknown which KSHV glycoprotein interacts with xCT. During infection of dendritic cells, macrophages and activated B cells, KSHV has been reported to utilize the C-type lectin DC-SIGN [43,44] via the highly mannosylated glycoprotein gB [45]. Crucial for the entry process of KSHV into endothelial cells and downstream signaling is the recently identified ephrin receptor tyrosine kinase A2 (EPHA2) at the host cell membrane [36,46] that binds the gH-gL dimer. Binding to EPHA2 follows KSHVs initial interaction with integrins, leading to EPHA2 phosphorylation, coordinated integrin-associated downstream signaling and endocytosis, which enables KSHV entry and subsequent infection [47]. 

Depending on the targeted cell type, KSHV has been reported to induce different routes of endocytic uptake, particularly macropinocytosis and clathrin-mediated endocytosis [32]. In endothelial cells, actin-dependent, dynamin-independent macropinocytosis has been described as a major route for productive KSHV infection [48]. This is in contrast to the route of entry in human fibroblasts, which occurs via dynamin-dependent, clathrin-mediated endocytosis [48,49]. Downstream of the described surface-mediated events, KSHV infection has been found to be dependent on activation of Focal adhesion kinase (FAK) [50], phosphorylated Src [51] and phosphoinositide 3-kinase (PI3K) signaling [52].

## 4. Human Papillomavirus

Papillomaviruses belong to the diverse family of *Papillomaviridae* comprising predominantly squamous-epitheliotropic, non-enveloped, small DNA viruses, which are ubiquitous throughout the world. There are more than 100 different types of human papillomaviruses (HPVs), which can be broadly divided into the types infecting mucosal epithelium and those infecting cutaneous epithelium [53]. HPV infections particularly affect squamous cells of the anogenital region, the skin and the upper aerodigestive tract. Depending on their ability to cause malignant cell transformation, HPVs have been grouped into low-risk and high-risk genotypes with HPV 16 and 18 being the most common oncogenic types world-wide. Indeed, HPVs are the most prevalent causes of sexually transmitted infections in the world and are known to be associated with the development of multiple carcinomas with virtually all cases of cervical cancer being attributable to infection by oncogenic HPVs [54].

HPV is generally not passed through body fluids but is rather transmitted by direct skin-to-skin contact. Viral infection predominantly occurs in the mitotically active basal layer of keratinocytes attached to the basement membrane of the host’s cutaneous and mucosal epithelia. While entry and infection in the upper epithelial layers has not been conclusively ruled out, cell cycle progression through early stages of mitosis was found to be critical for successful HPV infection providing a reason why HPV seems to infect only undifferentiated proliferating cells [55]. As the life cycle of HPV is dependent on the differentiation of basal cells into keratinocytes, the virus is highly tissue-restricted and thought to reach its cellular targets through disruptions of the integrity of the stratified or columnar epithelium that exposes the basal layer of cells for infection [56].

The icosahedral capsid coat of HPV containing the circular double-stranded DNA viral genome is composed of two structural proteins, the major capsid protein L1, and the minor capsid protein L2 which is mostly hidden from the capsid surface [57]. Although both L1 and L2, to different extents, are involved in the attachment of the virus to basal keratinocytes, the exact mechanism and receptors used by HPV to specifically bind to and infect epithelial cells is still highly debated. Differing experimental approaches and types of HPV particles used challenge the consistencies of the observations between studies [58]. The current two-step entry model entails that HPV, like many pathogens, initially binds to HSPGs at the epithelial cell surface or on the basement membrane which serve as primary attachment receptors [56,59,60,61,62]. Indeed, cells lacking HSPGs display greatly reduced HPV uptake [63,64,65]. In addition, laminin-5 secreted onto the extracellular matrix by keratinocytes has been proposed as a high-affinity initial attachment molecule [66]. The initial L1-mediated attachment is thought to induce conformational changes in the virus capsid through sequential engagement of multiple heparan sulfate (HS) binding sites eventually resulting in loss of affinity to the primary attachment receptor and transfer of the virus to an as yet unidentified secondary receptor [67]. The conformational changes in the virus capsid are thought to be brought about by cyclophilin B [68], thereby increasing the exposure of the otherwise hidden amino terminal portion of the L2 protein which contains a highly conserved consensus site for the proprotein convertase furin. Cleavage of L2 by furin is thought to be necessary for infection although it is debated whether it is a prerequisite at the early stage of binding and entry, or for endosome escape at later stages during the infection process [69,70]. Several secondary, predominantly L1-specific, receptors have been proposed, such as α6 integrin [71,72], EGFR and keratinocyte growth factor receptor (KGFR) [73] and tetraspanins [74]. Interestingly, the tetraspanin CD151 was found to be highly expressed in the basal layers of human cervical mucosa which—in association with integrins—may further explain the tissue-tropism of HPV16 infection [75]. Moreover, the annexin A2 heterotetramer (AIIt), a multifunctional protein involved in diverse cellular processes [76], has been implicated to act as an L2-specific receptor [77], regulating entry and intracellular trafficking of the virus [78]. 

Once bound to its specific uptake receptors, HPV enters the cell through a comparatively very slow asynchronous actin-dependent, and clathrin-, caveolin-, cholesterol-, and dynamin-independent endocytic process, with an average half-time of 12 h [79]. Internalization was found to be dependent on several signaling molecules such as EGFR, protein kinase C (PKC), p21-activated kinase 1 (PAK-1), the PI3K/Akt/mTOR pathway, and other yet unidentified tyrosine and serine/threonine kinases [79,80].

While there is no clear evidence for additive and/or alternative pathways, it is tempting to speculate that multiple internalization routes exist given the multitude of potential molecules involved in HPV binding and uptake. This is particularly interesting in the context of other viral entry pathways using similar entry molecules to HPV, for example cytomegalovirus (CMV) which infects cells via HSPGs (initial attachment), EGFR, integrins and/or annexin A2 (specific uptake) [81]; or Hepatitis C Virus (HCV) which employs HSPGs, EGFR, tetraspanins and similar intracellular signaling molecules ([82,83,84,85,86] and below). Moreover, the ability of different HPV types to species-specifically interact with individual binding and/or uptake molecules adds to the complexity of HPV entry and may provide an explanation of anatomical-site preferences of a given HPV type [87].

## 5. Merkel Cell Polyomavirus

Merkel cell polyomavirus (MCPyV) is the most recently identified member of the *Polyomaviridae* family which consists of non-enveloped, icosahedral, double-stranded DNA viruses capable of infecting a variety of species [88] including humans and having tumorigenic potential [88,89]. Merkel cell carcinoma (MCC) is generally found in sun-exposed areas of the skin, most commonly the head and neck [90]. MCPyV was first isolated from MCC tumors in 2008 [91] using Digital Transcriptome Subtraction [92]. Since then, a compelling line of evidence has been established for MCPyV as the causative agent of MCC, and 64%–88% of primary MCC tumors as well as metastases harbor MCPyV DNA and express MCPyV gene products [91,93,94,95,96,97,98,99,100]. MCC is a relatively rare, albeit severe, human cancer, predominantly presenting in elderly, Caucasian people [101] with suppressed immune systems and also HIV-infected individuals [102]. The incidence of MCC has drastically increased in the last two decades, presumably due to the increasing incidence of HIV/AIDS [90,101]. 

MCPyV is common in the population [99,103,104,105] and has a worldwide distribution [97,99,103,104,105,106,107]. MCPyV has been detected in children and seroprevalence found to gradually increase with age [105,106]. A correlation between siblings as well as mothers and small children was noted, suggesting transmission via close contact of skin and saliva during early childhood [106]. The virus has been detected in a diverse range of human tissues, although at significantly lower levels than in MCC tumors [108]. Keratinocytes are suspected to be the primary cells infected by MCPyV, supported by the chronic shedding of MCPyV from the skin [103]. However, the relationship between the cells that MCPyV infects and those that it transforms to give rise to MCC is currently unclear. MCC was thought to originate from Merkel cells due to similar expression patterns; however, this has come under debate due to differences in histomorphologic growth patterns and the discovery of markers previously thought to be of neuroendocrine cells, in a subset of lymophomas. A recent study proposes pro/pre- or pre-B cells as the cells of MCC due to the expression of terminal deoxynucleotidyl transferase (TdT) and paired box gene 5 (PAX5) in 72.8% and 100% of MCCs tested, respectively [109]. Jankowski *et al.* add that dysregulated expression of transcription factors in an epithelial cell could equally explain this expression pattern [110].

The MCPyV capsid consists of the structural proteins VP1 and VP2 in a ratio of 5:2 for native virions, while the VP3 minor capsid protein found in other polyomaviruses is undetectable in MCPyV [91,111]. Essential for MCPyV entry are pentameric knobs made up of the major capsid protein VP1 which bind to cellular receptors [112]. VP2 is essential for infectious entry in some cell types *in vitro* but others were transduced with MCPyV pseudovirions deficient in VP2. Knockdown of VP2 did not affect virion assembly, packaging of DNA or attachment to a target cell, indicating that the role of VP2 is in post-attachment entry. Myristoylation of VP2 has been shown to be important for infection of some cell types [111]. Current evidence points to a 2-step attachment-and-entry process for MCPyV whereby VP1 initially attaches to sulfated glycosaminoglycans, in particular heparan sulfate [113]. This is followed by transferal to a sialylated glycan which serves as a secondary post-attachment co-receptor and may mediate uptake by the cell. In concordance with the attachment mechanisms of other polyomaviruses [114,115,116,117], the ganglioside Gt1b was the first proposed receptor for MCPyV which was shown to interact with the sialic acid moieties on the Gt1b carbohydrate chain [118]. However, this was later disproved as Lec2 cells that lack sialylated glycans were found to bind MCPyV pseudovirions but were not productively infected [113]. The recently solved crystal structure of MCPyV major capsid protein VP1 showed a sialic acid binding site that, through mutation studies, was identified as having a role in post-attachment infection, not initial attachment. This sialic acid binding site differs from those of the previously characterized polyomaviruses BKPyV and murine polyomavirus for which sialylated glycans are primary attachment receptors and which is distinct from the yet unidentified binding site for sulfated glycosaminoglycans [112]. 

MCPyV seems to enter its target cells very slowly and asynchronously [119]. Eventually, viral replication takes place in the host cell nucleus, establishing productive infection. However, the events following penetration of the cell membrane and delivery of the encapsidated DNA to the nucleus are not yet fully established due to the lack of a reliable system to study MCPyV infection in cultured cells, especially with regard to infectivity and cell type tropism [119]. 

## 6. Hepatitis B Virus

Hepatitis B Virus (HBV) is the prototype of the *Hepadnaviridae* family of small, enveloped viruses that are tropic for the liver [120]. The virus is ubiquitous with a global distribution. HBV infection usually persists throughout the lifetime of the host as a result of noncytocidal infection of hepatocytes and their shedding of infectious virus into the blood stream and body fluids which represents the main route of transmission. Host immune response to the infection results in liver damage and scarring, creating a favorable environment for oncogenesis with a lifetime risk of developing liver cancer of 10%–25% in patients with chronic HBV infections [121]. HBV accounts for approximately 50% of hepatocellular carcinoma world-wide, which occurs mainly in less developed regions [122]. 

HBV is the smallest known enveloped virus. It is comprised of an icosahedral nucleocapsid containing the partially double-stranded, relaxed circular DNA (rcDNA) surrounded by a compact envelope [123,124]. The envelope consists of a host-derived cholesterol-rich lipid bilayer in which three surface glycoproteins, encoded by the same open reading frame, are embedded: L (large), M (middle) and S (small) [123,125,126]. The N-terminal preS1 domain of the L-protein is myristoylated at glycine 2 and this post-translational modification has been shown to be essential for viral infectivity [127,128,129]. Mutants lacking myristoylation are capable of normal viral assembly but are non-infectious to human hepatocytes [127,129]. 

Investigations into the entry mechanisms of HBV were hampered by the lack of a workable HBV infection system. Established human hepatoma cell lines were found to be unsusceptible to HBV and primary human hepatocyte cultures (PHHs) obtained from surgically resected liver pieces were susceptible but limited in availability, difficult to work with and variable in their preparation [130]. Primary hepatocyte cultures from the northern treeshrew *Tupaia belangeri* (PTH), which were shown to be susceptible to HBV, and a newly derived HepaRG hepatoma-derived cell line, provided the required infection systems to progress understanding of the mechanisms of HBV entry [131,132,133]. 

In 2005, Heparin Chromatography was reported as a method for purifying HBV virions from blood plasma, noting that heparin was a close homologue of liver heparan sulfate [134]. HSPGs were subsequently shown to act as an initial attachment receptor for HBV. The addition of heparin or dextran sulfate inhibits HBV infection, presumably by outcompeting HSPG for HBV binding, whereas a low-sulfated form of chondroitin sulfate does not [135]. Interestingly, liver-HS is also the binding receptor for other liver-targeting pathogens such as HCV (see below), dengue virus and malaria circumsporozoite [136,137,138]. 

Following attachment to HSPGs in the initial phase, HBV uptake through a specific entry receptor in the second phase of infection has long been elusive. While other viruses use a multitude of receptors for entry, only one candidate, namely sodium taurocholate co-transporting polypeptide (NTCP) [139,140,141], has been identified as a functional uptake receptor for HBV [139,140,141], initially using a synthetic preS1 construct together with PTH hepatocytes as target cells [139]. Importantly, susceptibility to HBV can be conferred on non-susceptible liver cell lines by transfecting them with NTCP [139,140,141]. NTCP is found exclusively in hepatocytes which explains the highly specific HBV tropism for the liver. 

HBV uptake into hepatocytes is thought to be regulated by clathrin-mediated endocytosis of the NTCP-HBV complex. PreS1 of the HBV L-protein interacts with clathrin heavy chain and clathrin adaptor protein AP-2. Employing sh-RNA knockdown of these molecules attenuated infection of a human primary hepatocyte cell line. In addition, treatment with an inhibitor of clathrin mediated endocytosis, chlorpromazine, inhibited HBV infection [142] presumably due to inhibition of NTCP endocytosis [143]. 

## 7. Hepatitis C Virus

Hepatitis C Virus (HCV) is a member of the *Flaviviridae* family—enveloped RNA viruses which are characterized by single-stranded RNA of positive polarity embedded in a capsid which is wrapped in a lipid and protein envelope. Like HBV, HCV is a hepatotropic virus and a major cause of chronic hepatitis, progressive liver disease and hepatocellular carcinoma worldwide. While about 3% of the global population is affected by HCV, approximately 1%–4% develop liver cancer, predominantly in Europe and Asia [122,144,145]. HCV is primarily transmitted through blood and infected blood products with very low risk of sexual or vertical transmission [146]. The virus can initiate infection of the host cell as cell-free particle and/or via cell-cell contact [147].

The viral envelope consists of host phosphatidylcholine, cholesterol-rich and triglyceride-rich lipoproteins incorporating host Very-Low Density Lipoprotein (VLDL) components such as apolipoproteins (Apos) AI, B, and E, as well as the viral glycoproteins E1 and E2 that form a heterodimer. These moeities are essential for infectivity of mature circulating HCV particles [148,149]. Unlike HBV entry as described above, infection of hepatocytes by HCV is more complex and involves multiple host cell molecules, suggesting a highly orchestrated process. However, initial attachment and concentrating of the virus to the hepatocyte surface is similar for both HBV and HCV and occurs via HSPGs which is mediated by the HCV glycoprotein E2 and host ApoE present in the viral envelope [136,150]. Subsequent internalization has been found to be mediated by a complex interplay between the HCV glycoproteins E1/E2 and several cellular factors which are important in defining HCV tissue specificity [151] including tetraspanin CD81, Scavenger Receptor B1 (SR-B1), and the tight junction proteins claudin-1 and occludin (reviewed in [152,153]). However, the sequential order of HCV-receptor interactions during the uptake phase is not well understood. It is hypothesized that the lipid-rich nature of the HCV particle may favor an initial interaction with SR-B1 possibly leading to conformational changes in the HCV glycoproteins [153], thereby contributing to HCV entry in an High Density Lipoprotein (HDL)-dependent manner [154,155]. SR-B1 is a lipoprotein receptor which is expressed primarily in liver and nonplacental steroidogenic tissues, and mediates both the selective uptake of cholesteryl esters and the efflux of cholesterol [156]. Moreover, several reports have indicated a role of SR-B1 in microbial recognition and uptake, such as that of *M. tuberculosis* [157] and *P. falciparum* [158]. While SR-B1 might function independently during intermediate steps of HCV infection by preparing the virus for hepatocyte entry, host surface molecules tetraspanin CD81 and claudin-1 form complexes that facilitate HCV uptake later in the entry process [159]. It is thought that HCV directly interacts with CD81 (and only indirectly with claudin-1 [160]) leading to further conformational rearrangement of the HCV E1/E2 glycoproteins [161]. Required for productive HCV infection is also the recently identified tight junction protein occludin [162,163,164], suggesting that HCV entry predominantly occurs at hepatocyte tight junctions [153]. Interestingly, Niemann-Pick C1-like cholesterol receptor (NPC1L1) [165] and the iron uptake receptor transferrin receptor 1 [166] have been assigned to play accessory but essential roles in HCV uptake. Lastly, the receptor tyrosine kinases EGFR and EPHA2 have recently been found to be involved in mediating HCV entry by regulating CD81-claudin-1 co-receptor associations [85,86], illustrating once more the complex involvement of multiple host surface molecules in HCV uptake. 

Following the interactions with the host receptors, HCV enters the cell via clathrin- and dynamin-dependent endocytic pathways [82,84] with downstream activation of Rho GTPase family members Rac, Rho and cell division cycle 42 (CDC42) and mitogen-activated protein kinase (MAPK) signaling cascades [83]. 

## 8. Human T Cell Lymphotropic Virus Type 1

Human T cell lymphotropic Virus Type-1 (HTLV-1) is a member of the *Retroviridae* family of single stranded RNA viruses that replicate in the host via reverse transcription [167,168,169]. As the etiological agent of Adult T cell lymphoma, HTLV-1 was independently described in the early 1980s [167,168,169,170] and became the first oncogenic retrovirus to be discovered. Chronic myelopathy and specific types of uveitis (HTLV-uveitis) have also since been associated with HTLV-1 infection [167,168,169,170,171,172].

HTLV-1 is endemic to south-western Japan [173] with a prevalence, in some areas, of >10% of the population [174] and is also prevalent in sub-Saharan Africa, the Caribbean, Iran and South America (reviewed in [175]). However, most HTLV-1 prevalence studies are based on screening of blood donors, pregnant women or sub-populations within endemic areas which introduces screening bias and limits an accurate estimation of number of infections which is thought to be greater than 20 million people [176]. 

HTLV-1 is transmitted by the introduction of cell-associated virus from mother to child through breast feeding [177], through sexual intercourse [178,179] or via blood or blood products [180]. The primary target cells of HTLV-1 are CD4^+^ T cells, however other cell types, such as dendritic cells [181], CD8^+^ T cells [182] and endothelial cells [183] can be infected. Cell-free virus is thought to be poorly infectious and is found at minimal levels in the peripheral blood even in individuals with a high percentage of T cells containing integrated virus [184]. Dendritic cells have, however, been shown to be susceptible to infection by cell-free virus and capable of rapidly transmitting it to T cells [181,185]. Cell-to-cell transmission accounts for the early spread of HTLV-1 in an individual. At a later stage, HTLV-1 infection persists through mitotic division of infected cells [186]. 

The HTLV-1 virion, like other retroviruses, is surrounded by a proteo-lipid envelope containing proteins with transmembrane (TM) and surface (SU) subunits. Most important for HTLV-1 infection is the product of the viral *env* gene which is cleaved by cellular proteases into the non-covalently linked TM subunit gp21 and the extracellular SU subunit gp46 [187], the latter consisting of an N-terminal Receptor-Binding Domain (RBD), a mid Proline rich region (PRR) and a C-terminal domain [187].

The current understanding of HTLV-1 entry into host cells is a multi-receptor model involving cellular HSPGs, NRP1 and glucose transporter-1 (GLUT-1) receptors through viral attachment and virus/cell fusion [188]. Initial attachment is mediated by interaction between the C-terminal domain of viral gp46 and HS moieties of cell surface HSPGs [189,190] of activated CD4^+^ T cells [191] or dendritic cells [181]. The length of HS chains has been found to affect the susceptibility of cells to HTLV-1 entry. Shorter HS chains facilitate closer attachment of the HTLV-1 virion to the target cell [192]. This eventually leads to viral binding to NRP1, a cell surface co-receptor for multiple growth factors such as vascular endothelial growth factor (VEGF), which can itself bind heparan sulfate [193,194,195]. The viral gp46 RBD contains the KPxR motif that has been identified as the NRP1 binding site [185]. The NRP1 b-domain which binds VEGF is required for HTLV-1 entry, and gp46 SU binding to NRP1 is inhibited by the HSPG binding domain of VEGF, indicating that HTLV-1 utilizes HSPG and NRP1 in complex by molecular mimicry of VEGF, particularly in cell types that normally lack VEGF production [185]. 

Subsequent to stable binding of HTLV-1 to HSPGs and NRP1, GLUT-1 binding sites on gp46 are exposed, presumably as a result of conformational changes in gp46 [188]. HTLV-1 binds GLUT-1 at its large extracellular loop (ECL1) [196,197]. The amino acid residues in the RBD of gp46 that are essential for GLUT-1 binding have been shown to be distinct from the binding sites of NRP1 and HSPGs, indicative of the formation of a multi-receptor complex [193,197]. Antibodies to GLUT-1 attenuate HTLV-1 fusion and infection but not binding to CD4^+^ T cells, indicating that binding to GLUT-1 is essential for fusion [196].

Cell-to-cell transmission requires cell contact between an infected cell and an uninfected cell and the formation of a Virological Synapse (VS) [198]. Igakura *et al.* observed that infected T cells formed conjugates with neighboring uninfected cells which induced polarization of the microtubule organization center (MTOC) to the point of contact [198]. Polarization of the MTOC is promoted by the interaction of intercellular adhesion molecule-1 or -3 (ICAM-1, ICAM-3) or vascular cell adhesion molecule-1 (VCAM-1) on infected cells with β-integrins, such as lymphocyte function-associated antigen-1 (LFA-1) on uninfected cells [199,200,201]. The HTLV-1 Env and Gag proteins and the HTLV-1 genome accumulate at the point of contact and then egress into the VS and interact with receptors on the conjugated, uninfected cell. NRP1 and GLUT-1 have been shown to co-localize at the point of contact and presumably promote the formation of the VS [193,202] which is also facilitated by the HTLV-1 encoded transcriptional transactivator protein Tax (Transcriptional Activator of pX region) [203]. Little is known about the mechanism at play to facilitate uptake of HTLV-1 subsequent to transmission across the VS, although it is speculated that this occurs via endocytosis of the virus as is the case for HIV [186]. 

## 9. Conclusion 

With the discovery of oncogenic viruses as the etiologic agents of various human cancers, targeting viral entry is particularly relevant in prevention of viral infection, antiviral therapy, vaccine development, and ultimately cancer prevention. The knowledge of the viral particle components and the host cell entry factors and their specific interactions can enable the design of efficient antiviral strategies. These strategies can include vaccines against distinct viral proteins required for viral entry or small molecules that target the host entry factors or the interactions between both. While almost all oncogenic viruses use HSPGs during initial attachment to their target cells, the specific uptake receptors differ considerably between the individual viruses (Table 1). This review summarized the current knowledge of the host factors required for oncogenic virus uptake in order to provide a concise overview of potential target molecules for prevention and/or treatment of oncogenic virus infection.

**Table 1:** Summary of cellular and molecular factors involved in human oncogenic virus uptake.

Virus	Infected cell type	Associated type of cancer	Attachment receptor(s)	Proposed uptake receptor(s) and/or co-receptors	Uptake mechanism
EBV	B cells, oropharyngeal epithelial cells	Burkitt’s, Hodgkin’s, immunosuppression-related, T and NK cell lymphomas, nasopharyngeal and stomach carcinomas	CD21 (CR2), CD35, β1 and α5 integrins	HLA class II molecules, αVβ6, αVβ8 integrins, NRP1	macropinocytosis and lipid raft-dependent endocytosis (epithelial cells)
KSHV	endothelial cells, B cells, monocytes (macrophages and dendritic cells), fibroblasts, epithelial cells	KS, B cell malignancies	HSPGs	α3β1, αVβ3, αVβ5, α9β1 integrins, xCT, DC-SIGN EPHA2	actin-dependent, dynamin-independent macro-pinocytosis (endothelial cells), dynamin-dependent, clathrin-mediated endocytosis (fibroblasts),
HPV	epithelial cells (particularly basal keratinocytes)	cervical, anal, head and neck cancer	HSPGs, laminin-5	α6 integrin, EGFR, KGFR, tetraspanins, AIIt	actin-dependent, clathrin- and lipid raft-independent endocytosis
MCPyV	keratinocytes	Merkel cell carcinoma	sulfated glycosamino-glycans (most likely HSPGs)	sialylated glycans	unknown
HBV	hepatocytes	hepatocellular carcinoma	HSPGs	NTCP	clathrin-mediated endocytosis
HCV	hepatocytes	hepatocellular carcinoma	HSPGs	tetraspanin CD81, SR-B1, claudin-1, occludin, NPC1L1, transferrin receptor 1, EGFR, EPHA2	clathrin- and dynamin-dependent endocytosis
HTLV-1	CD4^+^ T cells (dendritic cells, CD8^+^ T cells, endothelial cells)	Adult T-cell leukemia	HSPGs/NRP1	GLUT-1	viral synapse formation possibly followed by endocytosis

Listed are all known cellular factors involved in binding and uptake of the seven human tumor viruses described to date. Note that multiple molecules can be engaged by a particular virus in different combinations and not necessarily all at the same time. Please refer to the text for references.
